# Longitudinal Assessment of Cardiac Function After Craniospinal Irradiation in Pediatric Central Nervous System Tumor Survivors

**DOI:** 10.1016/j.jacadv.2026.102886

**Published:** 2026-06-15

**Authors:** Ana Carolina Izurieta-Pacheco, Alice Pozza, Emil Stefors, Marisa Signorile, Danielle R. Weidman, Farheen Ismail, Julie Bennett, Luc Mertens, Derek S. Tsang, Paul C. Nathan

**Affiliations:** a Division of Hematology/Oncology, Department of Pediatrics, The Hospital for Sick Children, Toronto, Ontario, Canada; b Oncology Department, Pediatric Cancer Center Barcelona, Hospital Sant Joan de Déu, Barcelona, Spain; c Division of Cardiology, Labatt Family Heart Centre, The Hospital for Sick Children, Toronto, Ontario, Canada; d Pediatric Cardiology Unit, Department of Women’s and Children’s Health, University of Padova, Padua, Italy; e Department of Paediatric Cardiology, Oslo University Hospital, Oslo, Norway; f Ted Rogers Computational Program, University Health Network, Toronto, Ontario, Canada; g Department of Radiation Oncology, Princess Margaret Cancer Centre, University Health Network, Toronto, Ontario, Canada; h Division of Medical Oncology and Hematology, Princess Margaret Cancer Centre, University Health Network, Toronto, Ontario, Canada

**Keywords:** cardiotoxicity, childhood cancer survivors, echocardiography, systolic dysfunction

## Abstract

**Background:**

Survival among children with central nervous system (CNS) tumors has improved markedly. However, long-term cardiac effects of craniospinal irradiation (CSI) remain poorly defined. Emerging data suggest an elevated risk for subclinical systolic dysfunction despite the absence of anthracycline exposure.

**Objectives:**

This study aimed to evaluate longitudinal echocardiographic measures of systolic and diastolic function following CSI and identify predictors of cardiac dysfunction.

**Methods:**

We conducted a retrospective, multi-institutional cohort study of pediatric patients diagnosed with a primary CNS tumor treated with CSI between January 2000 and September 2024. Echocardiographic assessments included M-mode left ventricular ejection fraction (LVEF), LV shortening fraction (LVSF), speckle-tracking global longitudinal strain (GLS), and Doppler-based diastolic indices. Longitudinal changes were modeled using linear mixed-effects models adjusted for demographic, treatment, cardiovascular, and endocrine variables.

**Results:**

Among 129 survivors (median age at diagnosis 8 years [IQR: 5-11]; at evaluation 23 years [IQR: 18-27]; median mean heart radiation dose 1,217 cGy [IQR: 2.340-3.600]), LVEF and LVSF declined progressively (*P* = 0.006 and *P* < 0.001). LVEF remained ≥50% in all patients, LVSF was <28% in 14 individuals. Diastolic parameters remained stable. GLS was assessed in 100 patients showing early post-CSI improvement followed by progressive decline (*P* < 0.001), 5 patients exhibited GLS <16%. Older age at diagnosis was associated with greater decline in LVEF and LVSF, while endocrine comorbidities correlated with lower LVEF as attained age increased. GLS trajectories differed by sex, with lower values in younger females.

**Conclusions:**

Progressive long-term decline in systolic function was observed in CSI-treated CNS tumor survivors despite modest cardiac radiation exposure. Age, sex, and endocrine comorbidities influence cardiac trajectories, supporting longitudinal cardiac surveillance.

Survival rates for pediatric cancer have improved substantially over recent decades, with current 5-year overall survival approaching 80%.^1^ As survival has increased, cardiovascular disease has emerged as the leading cause of long-term morbidity and premature mortality among childhood cancer survivors (CCS).^2^, ^3^, ^4^ Anthracycline chemotherapy and chest-directed radiation are well-recognized contributors to cardiotoxicity in CCS. Central nervous system (CNS) tumors represent the second most common group of pediatric malignancies after acute leukemia.^5^^,^^6^ Anthracyclines are not routinely used to treat CNS tumors given their poor penetration of the blood-brain barrier. Little is known about the long-term cardiac effects of craniospinal irradiation (CSI), a cornerstone therapy for many pediatric CNS tumors.^7^^,^^8^

Although CSI does not directly target the heart, scatter and exit-dose exposure with photon techniques can deliver approximately 28% to 50% of the prescribed spinal dose to cardiac structures.^9^ Cardiovascular risk may be further increased by concomitant exposure to alkylating agents, such as cyclophosphamide or ifosfamide.^8^^,^^10^ Endocrine late effects, including growth hormone deficiency, adrenal insufficiency, and the metabolic consequences of glucocorticoid replacement, may exacerbate this risk by promoting obesity, dyslipidemia, insulin resistance, and type 2 diabetes mellitus.^11^, ^12^, ^13^ The North American Childhood Cancer Survivor Study has reported that survivors of CNS tumors experience significantly increased risks of myocardial infarction, congestive heart failure, pericardial disease, and valvular dysfunction.^14^ Furthermore, echocardiographic assessments have demonstrated subclinical reductions in global longitudinal strain (GLS) measurements, even among survivors with preserved ejection fraction.^15^

Despite these findings, longitudinal data characterizing cardiac function trajectories after CSI remain limited.^16^ Current pediatric cardiotoxicity guidelines, which primarily address chest-directed radiation and anthracycline exposure, provide no specific recommendations for survivors treated with CSI.^17^ Defining the evolution of cardiac function over time in CSI-treated survivors is critical for identifying individuals at increased risk, refining surveillance protocols, and facilitating timely preventive or therapeutic strategies.

To address these gaps, we conducted a retrospective cohort study aimed at evaluating the longitudinal trajectories of cardiac function in CNS tumor survivors treated with CSI. By analyzing serial echocardiograms collected as part of routine care, we sought to identify systolic, diastolic, and GLS echocardiographic parameters affected following CSI and to determine clinical and treatment-related factors associated with declines in cardiac function.

## Methods

### Study design and setting

This retrospective, multi-institutional cohort study was conducted at The Hospital for Sick Children (Toronto, Canada) and Princess Margaret Cancer Centre, University Health Network (Toronto, Canada). The study received approval from the Research Ethics Boards of both institutions (REB #1000082138 and 23-5325).

### Study population

Patients were identified through the Hematology/Oncology Divisional database at The Hospital for Sick Children, with records for individuals who had transitioned to adult care cross-referenced at Princess Margaret Cancer Centre. Eligible participants were children and adolescents aged 0 to 18 years at the time of diagnosis of a primary CNS malignancy who received a CSI-containing regimen or thoracic spinal irradiation between January 2000 and September 2024. Inclusion criteria required at least one post-treatment echocardiogram with digitized images available for centralized review. Patients were excluded if they had preexisting cardiac disease, underlying genetic syndromes associated with cardiovascular abnormalities, or prior exposure to anthracycline chemotherapy. Follow-up extended from completion of CSI to the most recent echocardiographic assessment.

### Data sources and collection

Clinical data, including demographics, tumor characteristics, treatment details, and follow-up information, were extracted from institutional electronic health records. Radiation therapy parameters, including modality, prescribed dose, and mean heart dose, were obtained from the radiation planning system. Echocardiographic data were collected from clinical reports and supplemented by centralized review of digitized images by a pediatric cardiologist when necessary. Pre-CSI echocardiograms were rarely available and were therefore excluded. Chamber quantification measurements were performed in accordance with the American Society of Echocardiography pediatric guidelines.^18^ Left ventricular systolic function was assessed using the short axis M-mode view to calculate left ventricular ejection fraction (LVEF) and LVshortening fraction (LVSF); more contemporary two-dimensional (2D) or three-dimensional (3D) LVEF measurements were not consistently available across the cohort. Diastolic function was evaluated using pulsed Doppler of mitral inflow, with early (E) and late (A) velocities used to compute the E/A ratio, and tissue Doppler imaging of the lateral mitral annulus to derive the mitral E/E’ lateral ratio. GLS was calculated from 3 apical views (apical 4-chamber, apical 2-chamber, and apical 3-chamber views).^19^ However, GLS data were not routinely acquired in earlier years. Analyses involving GLS were restricted to studies in which GLS data were available (Supplemental Figure 1). Cardiovascular risk factors (dyslipidemia, overweight/obesity, diabetes mellitus, hypertension, chronic kidney disease, history of stroke) and treatment-related endocrinopathies were also abstracted to evaluate their associations with cardiac function.

### Statistical analyses

Statistical analyses were performed using R (version 4.0.3), with a 2-sided significance level of *P* < 0.05. Baseline clinical characteristics were summarized using descriptive statistics. Continuous variables were reported as medians with IQRs, and categorical variables as frequencies and proportions. Group differences were assessed using 2-sample *t*-tests or Mann-Whitney *U* tests for continuous variables, as appropriate, and Fisher exact tests for categorical variables.

Longitudinal echocardiographic parameters were analyzed using linear mixed-effects models with random patient-specific intercepts to account for within-subject correlation. All available data were included under a missing-at-random assumption. Time since completion of CSI was evaluated as time scale. To accommodate potential nonlinear patterns, time was modeled using natural cubic splines with 3 degrees of freedom. Model-based estimates were used to generate predicted trajectories with pointwise 95% CIs, and overall time effects were assessed using likelihood ratio tests. Decreasing number of observations at later follow-up is reflected by wider CIs. Individual trajectories for each echocardiographic parameter were visualized using spaghetti plots (Supplemental Figure 2) to display the underlying raw data and illustrate the magnitude and direction of the longitudinal change. To evaluate whether temporal trajectories differed by clinical or treatment-related exposures, time-by-exposure interaction terms were incorporated into multivariable linear mixed-effects models adjusted for age at diagnosis, sex, cumulative cyclophosphamide dose, mean heart dose, prior cardiac events during therapy, cardiovascular, and endocrine comorbidities, For dichotomous exposures, trajectories were visualized separately by group, and for continuous exposures, trajectories were displayed across low, medium, and high values corresponding to the 25th, 50th, and 75th percentiles to account for skewed variable distributions. The significance of interaction effects was assessed using likelihood ratio tests. Cumulative incidence of abnormal echocardiographic parameters was estimated using competing risk models with death as a competing event, and group differences were assessed using Gray’s tests. Administrative censoring was applied at 15 years of follow-up. Abnormal parameters were defined as LVEF <50%, LVSF <28%, and GLS <16%.

## Results

### Patient, tumor, and treatment characteristics

Among 187 patients treated with CSI for a CNS malignancy over the study period, 129 (69%) had echocardiographic data available and were therefore included in the study (Table 1). This subset differed from the full cohort, with higher-intensity therapy, higher mean heart doses, and greater prevalence of endocrine and cardiovascular comorbidities (Supplemental Table 1). The cohort was 59% male, with a median age of 8 years (IQR: 5-11) at diagnosis, and 23 years (IQR: 18-27) at the last follow-up. Medulloblastoma was the most common diagnosis (80%; n = 103), and 65% (n = 80) had nonmetastatic disease. Nearly all patients received CSI (98%; n = 127), with 2 patients (2%) receiving spine-only irradiation. Photon-based therapy was predominantly used (95%; n = 122). The median CSI dose was 2,360 cGy (IQR: 2,340-3,600), and the median mean heart dose was 1,217 cGy (IQR: 1,086-1,616). Most patients received cisplatin (91%; n = 118) and cyclophosphamide (79%; n = 102), and 50% (n = 64) underwent autologous hematopoietic stem cell transplantation. Cardiac events during therapy occurred in 21 patients (16%), most frequently hypertension (5%; n = 7) and pericardial effusion (5%; n = 6). Endocrine comorbidities were present in 67% (n = 87) of the cohort, while 28% (n = 36) had cardiovascular comorbidities, most commonly dyslipidemia (11%; n = 14) and overweight/obesity (6%, n = 8). Following completion of therapy, 16 patients (12%) experienced disease relapse and 2 patients (2%) developed a second malignant neoplasm. At last follow-up, 112 patients (87%) were alive and in remission, while 16 (12%) had died. All deaths were attributable to disease relapse. Use of cardiac medications was uncommon (2%; n = 3).

**Table 1:** Demographic and Clinical Characteristics of CNS Tumor Survivors With Echocardiographic Follow-Up

Sex, n = 129	
Female	53 (41%)
Male	76 (59%)
Age at diagnosis (y), n = 129	8 (5-11)
Current age (y), n = 129	23 (18-27)
Diagnosis, n = 129	
Atypical teratoid/rhabdoid tumor	2 (2%)
Embryonal rhabdomyosarcoma	1 (1%)
Ependymoma	4 (3%)
Germinoma	1 (1%)
Medulloblastoma	103 (80%)
Non-germinomatous germ cell tumor	4 (3%)
Oligodendroglioma (spinal cord)	1 (1%)
Pineoblastoma	10 (8%)
Primitive neuroectodermal tumor	3 (2%)
Disease stage, n =123	
Average risk	80 (65%)
High risk	43 (35%)
CSI as first-line therapy, n = 129	
Yes	124 (96%)
At relapse	5 (4%)
Radiation	
Radiotherapy location, n = 129	
Craniospinal	127 (98%)
Thoracic spine	2 (2%)
Radiation modality	
Photon	122 (95%)
Proton	7 (5%)
CSI dose (cGy), n = 129	2,340 (2,340-3,600)
CSI fractions, n = 129	13 (13-20)
Mean heart dose (cGy), n = 117	1,216.9 (1,086.3-1,615.9)
Chemotherapy, n = 129	
Any chemotherapy	124 (96%)
Type of chemotherapy, n = 129	
Vinca alkaloids	118 (91%)
Cisplatin	118 (91%)
Cyclophosphamide	102 (79%)
Ifosfamide	4 (3%)
Autologous HSCT	64 (50%)
Cardiac events during treatment, n = 129	21 (16%)
Type of cardiac event during treatment, n = 21	
Systolic dysfunction	3 (14%)
Hypertension	7 (33%)
Pericardial effusion	6 (29%)
Pulmonary edema	1 (5%)
Shock	3 (14%)
Arrhythmia	1 (5%)
Current patient status, n = 129	
Dead	16 (12%)
Remission	112 (87%)
Relapse	1 (1%)
Comorbidities, n = 129	
Endocrine	87 (67%)
Cardiovascular	36 (28%)
Type of endocrine comorbidities, n = 87	
Growth hormone deficiency	58 (67%)
Hypothyroidism	9 (10%)
Gonadal insufficiency	4 (5%)
Panhypopituitarism	14 (16%)
Other	2 (2%)
Type of cardiovascular comorbidities, n = 36	
Dyslipidemia	14 (39%)
Diabetes mellitus	6 (17%)
Hypertension	3 (8%)
Overweight/obesity	8 (22%)
Stroke	1 (3%)
Chronic kidney disease	4 (11%)
Cardiac medication use, n = 123	3 (2%)

Values are n (%) or median (IQR).

CSI = craniospinal irradiation; CNS = central nervous system; HSCT = hematopoietic stem cell transplantation.

### Cardiac function trajectories

**Figure 1:**
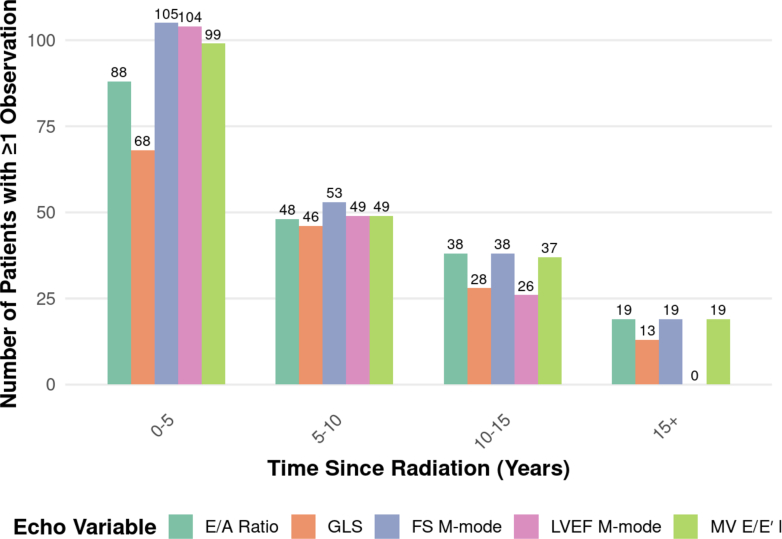
**Completeness of Echocardiographic Variables by Time Since Radiation Exposure** Bar graph showing the number of patients with ≥1 echocardiographic measurement by time since radiation exposure. Patients contributed a variable number of assessments over follow-up, with decreasing data availability at later time intervals. E/A ratio = early to late mitral inflow velocity ratio; GLS = global longitudinal strain; LVEF = left ventricular ejection fraction; MV E/E’ = mitral valve E to e’ ratio.

**Figure 2:**
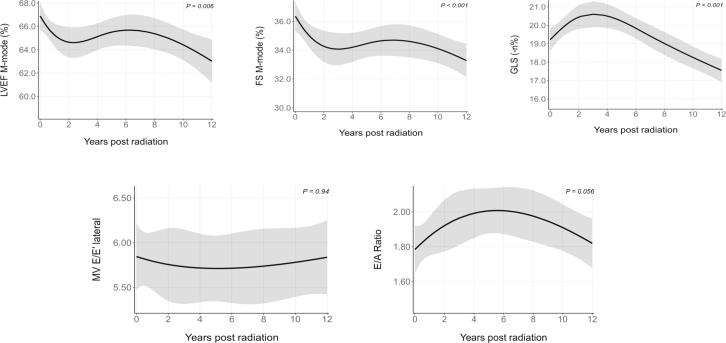
**Trajectories of Echocardiographic Parameters by Time Since Radiation Exposure** Modeled trajectories of LVEF (M-mode), LVSF (M-mode), GLS, MV E/E’ lateral, and E/A ratio by years postradiation. Lines represent model-estimated means; shaded areas denote 95% CIs; *P* values reflect overall trends. LVEF, LVSF, and GLS declined over time since radiation (LVEF *P* = 0.006; LVSF and GLS *P* < 0.001). MV E/E’ lateral remained unchanged across age and time. E/A ratio showed a borderline association with time since radiation (*P* = 0.056). LVSF = left ventricular shortening fraction; other abbreviations as in Figure 1.

**Figure 3:**
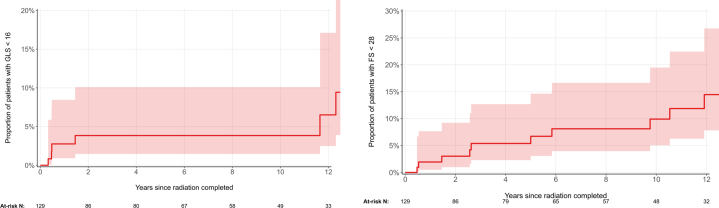
**Cumulative Incidence of Abnormal Echocardiographic Parameters Following Radiation Therapy** Cumulative incidence curves for abnormal echocardiographic parameters following radiation therapy, estimated using competing risk models with death as a competing event and administrative censoring at 12 years, GLS <16% (left) and LVSF <28% (right). Shaded areas represent 95% CIs; numbers at risk are shown below the x-axis. No patients developed LVEF <50% during follow-up; therefore, LVEF is not shown. Abbreviations as in Figures 1 and 2.

Patients contributed a variable number of echocardiographic assessments over follow-up (Supplemental Table 2). The number of patients contributing echocardiographic data decreased at later follow-up intervals (Figure 1). LVEF and LVSF declined significantly over time following radiation therapy (*P* = 0.006 and *P* < 0.001, respectively) (Figure 2); LVEF remained ≥50% in all patients, while LVSF fell below 28% in 14 patients (11%). Diastolic parameters remained stable over time. Mitral E/E’ lateral showed no significant change (*P* = 0.94), and the E/A ratio exhibited mild early increases until age 15 followed by slight late declines (*P* = 0.015). GLS demonstrated a biphasic pattern following radiation, with an early increase during the first 1 to 3 years followed by gradual decline with extended follow-up (*P* < 0.001). Five patients (4%) developed GLS <16%. The cumulative incidence of LVSF <28% increased from 3.0% at 2 years to 14.5% at 12 years following radiation therapy, whereas GLS <16% demonstrated a similarly progressive rise over time, reaching 6.5% at 12 years following radiation therapy (Table 2, Figure 3).

**Table 2:** Cumulative Incidence of Abnormal Echocardiographic Parameters by Time Since Radiation Therapy

Years Since Radiation Completion Parameter	CIF Estimates	
	GLS <16%	LVSF <28%
0	0.0% (0.0%, 0.0%)	0.0% (0.0%, 0.0%)
2	3.8% (1.5%, 10.1%)	3.0% (1.0%, 9.2%)
4	3.8% (1.5%, 10.1%)	5.4% (2.3%, 12.7%)
6	3.8% (1.5%, 10.1%)	8.1% (4.0%, 16.6%)
8	3.8% (1.5%, 10.1%)	8.1% (4.0%, 16.6%)
10	3.8% (1.5%, 10.1%)	9.9% (5.0%, 19.5%)
12	6.5% (2.5%, 17.1%)	14.5% (7.8%, 26.7%)

CIF = cumulative incidence function; GLS = global longitudinal strain; LVSF = left ventricular shortening fraction.

**Figure 4:**
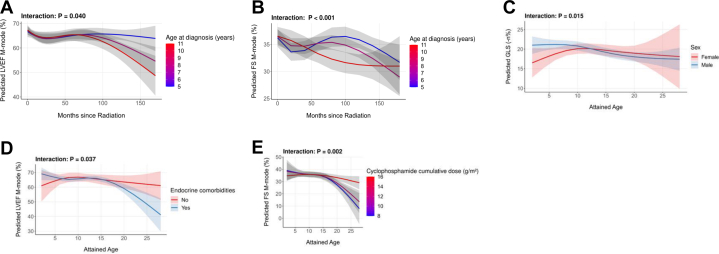
**Effect Modification of Echocardiographic Trajectories** Predicted trajectories from adjusted multivariable models demonstrating effect modification of systolic function over time. (A) LVEF (M-mode) by age at diagnosis across months since radiation (interaction *P* = 0.041). (B) LVSF (M-mode) by age at diagnosis across months since radiation (interaction *P* < 0.001). (C) GLS by sex across attained age (interaction *P* = 0.015). (D) LVEF (M-mode) by endocrine comorbidities across attained age (interaction *P* = 0.037). (E) LVSF (M-mode) by cyclophosphamide cumulative dose across attained age (interaction *P* = 0.002). Shaded areas represent 95% CIs. Abbreviations as in Figures 1 and 2.

In multivariable models adjusted for age at diagnosis, sex, cumulative cyclophosphamide dose, mean heart dose, cardiac events during therapy, cardiovascular and endocrine comorbidities (Figure 4, Tables 3 and 4, Supplemental Table 3), older age at diagnosis was associated with greater declines in LVEF and LVSF over time (*P* = 0.040 and *P* < 0.001, respectively). Endocrine comorbidities were linked to progressively lower LVEF as attained age increased (*P* = 0.037). LVSF trajectories were influenced by cumulative cyclophosphamide exposure, with higher cumulative doses associated with relative stability, while patients who received mid-to-lower doses experienced more pronounced declines after age 15 (*P* = 0.002). GLS trajectories also differed by sex, with females exhibiting lower absolute GLS values at younger ages compared with males (*P* = 0.015), although these differences attenuated during adolescence. Cardiovascular comorbidities did not significantly modify GLS patterns. Diastolic parameters were not significantly influenced by treatment exposures or comorbidities, and no associations were observed between mean heart dose and any echocardiographic measures.

**Table 3:** Multivariable Mixed-Effects Analyses of Longitudinal Left Ventricular Ejection Fraction: M-mode

	Model∗	Cyclophosphamide Cumulative Dose β (95% CI)	*P* Value	Mean Heart Dose β (95% CI)	*P* Value	Male β (95% CI)	*P* Value	Cardiac Event During Treatment β (95% CI)	*P* Value	Endocrine Comorbidities β (95% CI)	*P* Value	Cardiovascular Comorbidities β (95% CI)	*P* Value	Age at Diagnosis β (95% CI)	*P* Value
Age at diagnosis	T	0.01 (−0.14, 0.15)	0.92	−0.13 (−1.10, 0.83)	0.78	−0.15 (−1.76, 1.46)	0.85	−0.35 (−2.41, 1.71)	0.74	0.13 (−2.13, 2.38)	0.91	−0.07 (−1.93, 1.80)	0.94		
	A	0.01 (−0.13, 0.15)	0.91	−0.04 (−0.99, 0.92)	0.94	−0.45 (−2.03, 1.12)	0.58	−0.61 (−2.68, 1.46)	0.57	−0.07 (−2.30, 2.17)	0.95	−0.03 (−1.89, 1.83)	0.97		
Cyclophosphamide cumulative dose	T			−0.17 (−1.14, 0.80)	0.73	−0.31 (−1.84, 1.23)	0.70	0.17 (−1.91, 2.24)	0.88	−0.02 (−2.23, 2.20)	0.99	−0.66 (−2.34, 1.02)	0.45	−0.19 (−0.43, 0.05)	0.130
	A			0.03 (−0.98, 1.04)	0.96	−0.34 (−1.95, 1.27)	0.68	−0.46 (−2.66, 1.73)	0.68	−0.12 (−2.41, 2.18)	0.92	−0.31 (−2.08, 1.47)	0.74	0.03 (−0.26, 0.33)	0.83
Mean heart dose	T	−0.03 (−0.17, 0.11)	0.66			0.01 (−1.58, 1.59)	0.99	0.26 (−1.77, 2.29)	0.80	0.15 (−2.06, 2.36)	0.89	−0.56 (−2.27, 1.15)	0.52	−0.24 (−0.49, 0.01)	0.066
	A	0.00 (−0.13, 0.14)	0.95			−0.21 (−1.81, 1.38)	0.79	−0.29 (−2.33, 1.75)	0.78	0.04 (−2.19, 2.27)	0.97	−0.32 (−2.09, 1.44)	0.72	0.03 (−0.26, 0.32)	0.83
Sex	T	−0.01 (−0.14, 0.13)	0.94	−0.17 (−1.09, 0.75)	0.72	−1.54 (−4.28, 1.20)	0.27	−0.13 (−2.10, 1.85)	0.90	−0.14 (−2.34, 2.06)	0.90	−0.71 (−2.40, 0.99)	0.42	−0.23 (−0.47, 0.01)	0.066
	A	−0.01 (−0.14, 0.13)	0.94	−0.19 (−1.11, 0.74)	0.70	−1.74 (−7.93, 4.45)	0.58	−0.32 (−2.35, 1.71)	0.76	−0.34 (−2.54, 1.86)	0.76	−0.58 (−2.32, 1.16)	0.52	0.02 (−0.28, 0.31)	0.92
Cardiac event during treatment	T	−0.02 (−0.15, 0.12)	0.80	−0.18 (−1.12, 0.75)	0.70	−0.12 (−1.67, 1.44)	0.88	−0.64 (−3.93, 2.66)	0.70	−0.05 (−2.26, 2.16)	0.96	−0.57 (−2.28, 1.13)	0.51	−0.23 (−0.48, 0.01)	0.067
	A	−0.01 (−0.14, 0.12)	0.89	−0.16 (−1.06, 0.73)	0.72	−0.09 (−1.60, 1.42)	0.91	−7.15 (−13.48, −0.83)	0.028	−0.92 (−3.10, 1.27)	0.41	−0.60 (−2.27, 1.07)	0.48	−0.03 (−0.31, 0.26)	0.86
Endocrine comorbidities	T	−0.01 (−0.14, 0.13)	0.92	−0.17 (−1.09, 0.75)	0.72	−0.09 (−1.62, 1.45)	0.91	0.04 (−1.92, 2.01)	0.96	−0.41 (−3.68, 2.87)	0.81	−0.57 (−2.27, 1.13)	0.51	−0.24 (−0.48, 0.01)	0.062
	A	−0.00 (−0.14, 0.13)	0.98	−0.20 (−1.14, 0.73)	0.67	−0.25 (−1.83, 1.32)	0.75	−0.09 (−2.20, 2.03)	0.93	6.16 (−2.68, 15.01)	0.174	−0.53 (−2.26, 1.20)	0.55	−0.04 (−0.35, 0.26)	0.79
Cardiovascular comorbidities	T	−0.01 (−0.15, 0.13)	0.89	−0.20 (−1.12, 0.72)	0.67	−0.16 (−1.70, 1.38)	0.84	0.07 (−1.90, 2.04)	0.94	−0.12 (−2.32, 2.08)	0.92	−1.27 (−4.36, 1.81)	0.42	−0.24 (−0.48, 0.00)	0.055
	A	0.00 (−0.14, 0.14)	1.00	−0.20 (−1.13, 0.72)	0.67	−0.26 (−1.84, 1.32)	0.75	−0.36 (−2.39, 1.67)	0.73	−0.42 (−2.61, 1.77)	0.71	0.55 (−9.48, 10.59)	0.91	−0.00 (−0.29, 0.28)	0.98

β coefficients represent adjusted associations from multivariable linear mixed-effects models with patient-level random intercepts. Time was modeled using natural cubic splines. All models account for repeated measures and adjust for the covariates shown in each column. Cyclophosphamide dose and mean heart dose are continuous variables; sex and clinical comorbidities are binary (reference: female and absence of condition, respectively). 95% CIs are shown in brackets; *P* values are two-sided.

T = time since craniospinal irradiation and A = attained age (years).

∗ Two time scales were used.

**Table 4:** Multivariable Mixed-Effects Analyses of Longitudinal Left Ventricular Ejection Fraction: Global Longitudinal Strain

	Model∗	Cyclophosphamide Cumulative Dose β (95% CI)	*P* Value	Mean Heart Dose β (95% CI)	*P* Value	Male β (95% CI)	*P* Value	Cardiac Event During Treatment β (95% CI)	*P* Value	Endocrine Comorbidities β (95% CI)	*P* Value	Cardiovascular Comorbidities β (95% CI)	*P* Value	Age at Diagnosis β (95% CI)	*P* Value
Age at diagnosis	T	−0.02 (−0.09, 0.06)	0.63	−0.02 (−0.50, 0.45)	0.92	0.48 (−0.35, 1.32)	0.26	−0.28 (−1.32, 0.75)	0.59	−0.82 (−2.00, 0.35)	0.174	−0.29 (−1.24, 0.66)	0.55		
	A	−0.01 (−0.09, 0.06)	0.73	0.03 (−0.46, 0.52)	0.90	0.49 (−0.37, 1.35)	0.27	−0.30 (−1.37, 0.77)	0.59	−0.46 (−1.68, 0.77)	0.47	−0.21 (−1.19, 0.78)	0.68		
Cyclophosphamide cumulative dose	T			−0.03 (−0.57, 0.50)	0.91	0.49 (−0.40, 1.37)	0.29	−0.56 (−1.75, 0.62)	0.36	−0.63 (−1.85, 0.58)	0.31	−0.03 (−0.98, 0.92)	0.95	−0.26 (−0.40, −0.12)	<0.001
	A			0.07 (−0.48, 0.62)	0.80	0.37 (−0.55, 1.29)	0.44	−0.46 (−1.73, 0.80)	0.47	−0.09 (−1.40, 1.22)	0.89	−0.36 (−1.36, 0.64)	0.48	−0.03 (−0.20, 0.14)	0.71
Mean heart dose	T	−0.00 (−0.07, 0.07)	0.93			0.70 (−0.17, 1.56)	0.120	−0.14 (−1.18, 0.89)	0.79	−0.36 (−1.50, 0.78)	0.54	−0.10 (−1.00, 0.80)	0.83	−0.21 (−0.34, −0.08)	0.003
	A	−0.02 (−0.09, 0.06)	0.65			0.41 (−0.47, 1.29)	0.37	−0.03 (−1.11, 1.05)	0.96	0.09 (−1.10, 1.28)	0.88	−0.23 (−1.17, 0.71)	0.64	−0.02 (−0.18, 0.14)	0.80
Sex	T	0.01 (−0.06, 0.08)	0.76	−0.14 (−0.60, 0.32)	0.55	1.24 (−0.17, 2.65)	0.086	−0.30 (−1.30, 0.69)	0.56	−0.88 (−1.99, 0.22)	0.121	−0.02 (−0.88, 0.85)	0.97	−0.27 (−0.40, −0.14)	<0.001
	A	−0.02 (−0.09, 0.06)	0.68	0.05 (−0.43, 0.53)	0.84	3.99 (0.54, 7.44)	0.025	−0.44 (−1.52, 0.63)	0.42	−0.38 (−1.55, 0.79)	0.53	−0.18 (−1.12, 0.76)	0.71	−0.09 (−0.24, 0.06)	0.26
Cardiac event during treatment	T	−0.02 (−0.09, 0.05)	0.65	−0.05 (−0.51, 0.42)	0.84	0.46 (−0.37, 1.30)	0.28	−1.62 (−3.39, 0.15)	0.076	−0.70 (−1.83, 0.43)	0.23	−0.01 (−0.92, 0.90)	0.98	−0.26 (−0.40, −0.13)	<0.001
	A	−0.04 (−0.12, 0.04)	0.31	0.21 (−0.28, 0.71)	0.40	0.37 (−0.52, 1.26)	0.42	−0.58 (−3.87, 2.70)	0.73	−0.27 (−1.47, 0.94)	0.67	−0.29 (−1.25, 0.67)	0.56	−0.05 (−0.21, 0.10)	0.52
Endocrine comorbidities	T	−0.00 (−0.07, 0.07)	0.98	−0.07 (−0.53, 0.40)	0.78	0.42 (−0.41, 1.24)	0.33	−0.41 (−1.43, 0.61)	0.43	−1.01 (−2.52, 0.50)	0.191	−0.09 (−0.99, 0.80)	0.84	−0.24 (−0.37, −0.10)	<0.001
	A	−0.02 (−0.09, 0.06)	0.65	0.11 (−0.39, 0.61)	0.67	0.28 (−0.60, 1.16)	0.53	−0.48 (−1.62, 0.65)	0.41	−3.23 (−7.52, 1.05)	0.142	−0.13 (−1.09, 0.82)	0.78	−0.06 (−0.23, 0.10)	0.46
Cardiovascular comorbidities	T	0.00 (−0.07, 0.07)	0.96	−0.06 (−0.53, 0.40)	0.80	0.39 (−0.44, 1.22)	0.36	−0.32 (−1.35, 0.71)	0.54	−0.80 (−1.94, 0.33)	0.168	−0.59 (−2.09, 0.91)	0.44	−0.27 (−0.40, −0.13)	<0.001
	A	−0.03 (−0.10, 0.04)	0.43	0.17 (−0.32, 0.66)	0.50	0.31 (−0.57, 1.19)	0.49	−0.51 (−1.60, 0.59)	0.37	−0.37 (−1.56, 0.82)	0.54	0.30 (−4.23, 4.83)	0.90	−0.05 (−0.20, 0.11)	0.56

β coefficients represent adjusted associations from multivariable linear mixed-effects models with patient-level random intercepts. Time was modeled using natural cubic splines. All models account for repeated measures and adjust for the covariates shown in each column. Cyclophosphamide dose and mean heart dose are continuous variables; sex and clinical comorbidities are binary (reference: female and absence of condition, respectively). 95% CIs are shown in brackets; *P* values are 2-sided.

T = time since craniospinal irradiation and A = attained age (years).

∗ Two time scales were used.

## Discussion

In this multi-institutional cohort of childhood CNS tumor survivors treated with CSI, we identified longitudinal declines in left ventricular systolic function despite modest mean heart radiation exposure and the absence of anthracycline therapy. Specifically, we observed: 1) progressive declines in LVEF and LVSF over time since CSI. Although these declines were statistically significant, systolic function remained largely preserved with LVEF ≥50% in all patients, while 11% developed LVSF <28%, consistent with subclinical systolic impairment; 2) GLS demonstrated a biphasic trajectory, with early postradiation increases followed by gradual decline over extended follow-up; overt abnormalities were uncommon, with only 4% of patients developing GLS <16%; and 3) modulation of these trajectories by clinical and treatment-related factors, including older age at diagnosis, female sex, cyclophosphamide exposure, and endocrine comorbidities. Collectively, these findings provide novel insights into subclinical myocardial remodeling among CSI-treated survivors and address an important gap in cardio-oncology survivorship research (Central Illustration).

**Central Illustration:**
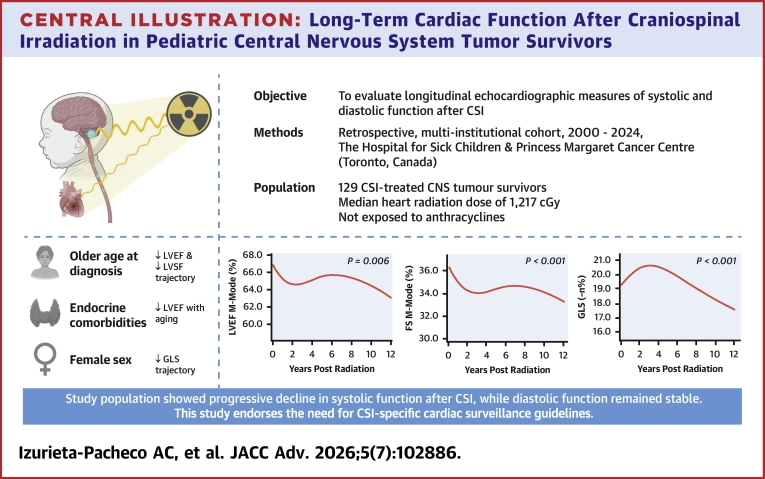
**Long-Term Cardiac Function After Craniospinal Irradiation in Pediatric Central Nervous System Tumor Survivors** Longitudinal trajectories of left ventricular systolic function (LVEF, LVSF) and GLS show progressive declines over time despite low mean heart radiation doses, whereas diastolic function remains stable. Age at diagnosis, sex, and endocrine comorbidities modify cardiac trajectories. CNS = central nervous system; CSI = craniospinal irradiation; other abbreviations as in Figures 1 and 2.

Prior cardio-oncology studies have focused predominantly on anthracycline-related cardiotoxicity, with limited data on long-term cardiac outcomes following CSI alone.^20^^,^^21^ Dosimetric analyses consistently demonstrate low-to-moderate scatter radiation exposure to the heart, with lower doses observed using contemporary proton therapy.^22^^,^^23^ Accordingly, current survivorship guidelines generally do not recommend routine cardiac imaging for CCS who received <15 Gy mean heart dose in the absence of anthracycline therapy.^24^^,^^25^ Our findings challenge the assumption that such low-dose cardiac radiation is benign. Despite a low median mean heart dose of 1,217 cGy, progressive declines in LVEF and LVSF, alongside evolving abnormalities in myocardial deformation were observed. The absence of a clear dose-response relationship suggests that other patient- and treatment-specific factors may also influence heart function after cancer treatment.

Speckle-tracking echocardiography with assessment of GLS has emerged as a sensitive measure of myocardial function, detecting early changes prior to declines in LVEF.^26^ In noncancer populations, GLS has demonstrated stronger prognostic value than LVEF for predicting mortality and major adverse cardiac events.^27^ In CCS, prior studies have shown that a substantial subset exhibits abnormal GLS despite preserved LVEF, reflecting subclinical myocardial dysfunction. For instance, Martinez et al^15^ reported lower GLS in CSI-treated patients compared with age-matched controls (>12 months post-CSI: −16.2% ± 5.4% vs −21.6% ± 3.7%), while Armstrong et al^28^ found that 28% of CCS with normal 3D LVEF had abnormal GLS. Our cohort demonstrated a pattern of early postradiation GLS increase followed by gradual decline, though overt GLS abnormalities were uncommon.

The observed age-dependent declines in LVEF and LVSF align with patterns of subclinical ventricular dysfunction previously described in CCS exposed to chest or mediastinal radiotherapy, in which functional deficits may remain clinically silent for years.^20^^,^^29^ This progression reflects the pathophysiological cascade of radiation-induced injury, including microvascular damage, fibrosis, and reduced myocardial reserve.^30^ Population-level studies have demonstrated that even low-to-moderate radiotherapy doses across cardiac volumes increase the long-term risk of serious cardiac events.^21^ Additionally, radiation exposure to specific cardiac substructures, including the left ventricle or coronary arteries, has been linked to heightened risk of heart failure, coronary artery disease, and valvular dysfunction.^31^

Older age at diagnosis was independently associated with greater declines in LVEF and LVSF. Unlike anthracycline cardiotoxicity where younger age often confers increased vulnerability, the impact of age at radiation exposure may differ.^21^^,^^32^, ^33^, ^34^ A few plausible explanations warrant consideration. First, physiological data demonstrate that myocardial structure, metabolic programming, and overall cardiovascular geometry continue evolving through childhood and adolescence, suggesting that the developmental stage at the time of exposure may modulate vulnerability to later cardiac injury.^35^ Second, older children may have received higher cumulative treatment intensity, or be more prone to early metabolic or endocrine impairments, which could synergize with radiation to accelerate myocardial damage.^36^^,^^37^

Endocrine late effects, including pituitary, hypothalamic, and metabolic disorders, are common among CNS tumor survivors treated with CSI.^38^, ^39^, ^40^ In our cohort, these comorbidities were present in approximately two-thirds of patients and independently associated with progressive declines in LVEF with increasing attained age. Endocrine dysfunction has been linked to adverse metabolic and cardiovascular risk profiles, including dyslipidemia, insulin resistance, central adiposity, and hypertension, which may predispose to long-term vascular and myocardial injury.^40^, ^41^, ^42^ However, data directly connecting these disorders to subclinical myocardial remodeling or progressive ventricular dysfunction remain limited.

Sex-specific differences in myocardial function are well-documented in adults, suggesting that biological sex may influence cardiac function.^43^^,^^44^ Data in pediatric populations are limited; in our cohort, females exhibited lower absolute GLS at younger ages compared with males, although these differences attenuated during adolescence. Cyclophosphamide exposure demonstrated an unexpected pattern, with greater LVSF declines observed in patients who received lower cumulative doses, likely reflecting confounding or treatment differences rather than a true dose-response effect.

### Study limitations

While this study provides valuable longitudinal insights into cardiac function after CSI, some limitations merit consideration. The retrospective design and absence of baseline pre-CSI echocardiograms limit characterization of early therapy-related cardiac changes. Echocardiographic assessments were collected over 2 decades across 2 centers, introducing potential heterogeneity and reflecting evolving treatment protocols over time. Notably, GLS was not available at all time points and was predominantly measured in more recent studies. No retrospective offline strain analyses were performed on earlier studies. Additionally, LVEF assessments relied primarily on M-mode, a less contemporary method. Simpson’s biplane 2D or 3D LVEF measurements were not consistently available across the cohort. More comprehensive guideline-based echocardiographic parameters for diastolic function assessment were not uniformly available across the study period. Comparisons of longitudinal trajectories between survivors and nonsurvivors were limited, as most patients who died had only a single early echocardiographic assessment. The majority of patients who underwent autologous hematopoietic stem cell transplantation were treated according to the SJMB03 protocol, which was the institutional standard-of-care protocol during part of the study period, though it is no longer widely used. Younger children were underrepresented due to radiation-sparing approaches, which may influence observed age-related associations. Residual confounding from unmeasured variables, such as lifestyle factors, cannot be excluded. Proton therapy was delivered outside of Canada, and detailed proton treatment planning data were unavailable. Finally, the predominance of photon-based CSI may limit generalizability to patients treated with contemporary proton therapy, which may reduce or avoid cardiotoxicity given its ability to treat the neuraxis while sparing the heart completely.^45^ This is an area that warrants further study in the future to confirm this modality confers lower risk of cardiotoxic late effects. Importantly, this cohort remains relatively young, and whether some individuals will progress to clinically overt cardiac disease with advancing age remains unknown.

## Conclusions

Childhood CNS tumor survivors treated with CSI exhibited progressive declines in left ventricular systolic function and GLS over long-term follow-up, despite modest cardiac radiation doses and absence of anthracycline exposure. Age at diagnosis, sex, and endocrine comorbidities influenced the magnitude and trajectory of these changes, whereas diastolic function remained stable. These findings indicate that in patients treated with CSI, subclinical myocardial remodeling may occur, providing novel insights into the cardiac sequelae of CSI in CNS tumor survivors. However, longer follow-up is needed to clarify the clinical implications of these subclinical changes and to determine whether current cardiac surveillance guidelines should be adapted to better identify and manage this at-risk population.

Perspectives **COMPETENCY IN MEDICAL KNOWLEDGE:** Childhood CNS tumor survivors treated with CSI exhibit progressive, subclinical declines in left ventricular systolic function despite low cardiac radiation doses and no anthracycline exposure, challenging current assumptions about cardiotoxic risk. Age at diagnosis, sex, and endocrine comorbidities influence cardiac function trajectories post-CSI.

**TRANSLATIONAL OUTLOOK:** Future studies should determine optimal surveillance intervals, validate risk-adapted screening strategies, and assess whether early detection of subclinical dysfunction improves long-term cardiovascular outcomes.

### Declaration of Generative AI and AI-Assisted Technologies in the Writing Process

AI software (ChatGPT) was used solely for minor language and spelling corrections to assist with English clarity, as English is not the corresponding author’s first language. No content, analysis, or conclusions were generated by AI.

## Funding support and author disclosures

Funded, in part, by a 10.13039/501100000024Canadian Institutes of Health Research (CIHR) Foundation Grant (PI: Dr Nathan). Drs Tsang and Bennett are consultants with Need (https://www.need.ai), outside the submitted work. All other authors have reported that they have no relationships relevant to the contents of this paper to disclose.

## References

[1] Robison L.L., Hudson M.M. (2014). Survivors of childhood and adolescent cancer: life-long risks and responsibilities. Nat Rev Cancer.

[2] Mertens A.C., Liu Q., Neglia J.P. (2008). Cause-specific late mortality among 5-Year survivors of childhood cancer: the childhood cancer survivor study. JNCI J Natl Cancer Inst.

[3] Armstrong G.T., Liu Q., Yasui Y. (2009). Late mortality among 5-Year survivors of childhood cancer: a summary from the childhood cancer survivor study. J Clin Oncol.

[4] Armenian S.H., Armstrong G.T., Aune G. (2018). Cardiovascular disease in survivors of childhood cancer: insights into epidemiology, pathophysiology, and prevention. J Clin Oncol.

[5] Steliarova-Foucher E., Colombet M., Ries L.A.G. (2017). International incidence of childhood cancer, 2001–10: a population-based registry study. Lancet Oncol.

[6] Siegel D.A., King J.B., Lupo P.J. (2023). Counts, incidence rates, and trends of pediatric cancer in the United States, 2003-2019. JNCI J Natl Cancer Inst.

[7] Leerink J.M., De Baat E.C., Feijen E.A.M. (2020). Cardiac disease in childhood cancer survivors. JACC CardioOncol.

[8] Feijen E.A.M.L., Font-Gonzalez A., Van Der Pal H.J.H. (2019). Risk and temporal changes of heart failure among 5-Year childhood cancer survivors: a DCOG-LATER study. J Am Heart Assoc.

[9] Chojnacka M., Skowrońska-Gardas A., Morawska-Kaczyńska M., Zygmuntowicz-Piętka A., Pędziwiatr K., Semaniak A. (2010). Craniospinal radiotherapy in children: electron- or photon-based technique of spinal irradiation. Rep Pract Oncol Radiother.

[10] Rosa G.M., Gigli L., Tagliasacchi M.I. (2016). Update on cardiotoxicity of anti-cancer treatments. Eur J Clin Invest.

[11] Cooksey R., Wu S.Y., Klesse L. (2019). Metabolic syndrome is a sequela of radiation exposure in hypothalamic obesity among survivors of childhood brain tumors. J Invest Med.

[12] Van Schaik J., Van Roessel I.M.A.A., Schouten-van Meeteren N.A.Y.N. (2021). High prevalence of weight gain in childhood brain tumor survivors and its association with hypothalamic-pituitary dysfunction. J Clin Oncol.

[13] Pietilä S., Mäkipernaa A., Sievänen H., Koivisto A., Wigren T., Lenko H.L. (2009). Obesity and metabolic changes are common in young childhood brain tumor survivors. Pediatr Blood Cancer.

[14] Mulrooney D.A., Yeazel M.W., Kawashima T. (2009). Cardiac outcomes in a cohort of adult survivors of childhood and adolescent cancer: retrospective analysis of the Childhood Cancer Survivor Study cohort. BMJ.

[15] Martinez H.R., Salloum R., Wright E. (2021). Echocardiographic myocardial strain analysis describes subclinical cardiac dysfunction after craniospinal irradiation in pediatric and young adult patients with central nervous system tumors. Cardio-Oncol.

[16] Ryan T.D., Bates J.E., Kinahan K.E. (2025). Cardiovascular toxicity in patients treated for childhood cancer: a scientific statement from the American heart Association. Circulation.

[17] Mertens L., Singh G., Armenian S. (2023). Multimodality imaging for cardiac surveillance of cancer treatment in children: recommendations from the American Society of Echocardiography. J Am Soc Echocardiogr.

[18] Lopez L., Saurers D.L., Barker P.C.A. (2024). Guidelines for performing a comprehensive pediatric transthoracic echocardiogram: recommendations from the American Society of echocardiography. J Am Soc Echocardiogr.

[19] Badano L.P., Kolias T.J., Muraru D. (2018). Standardization of left atrial, right ventricular, and right atrial deformation imaging using two-dimensional speckle tracking echocardiography: a consensus document of the EACVI/ASE/Industry task force to standardize deformation imaging. Eur Heart J Cardiovasc Imaging.

[20] Mulrooney D.A., Armstrong G.T., Huang S. (2016). Cardiac outcomes in adult survivors of childhood cancer exposed to cardiotoxic therapy: a cross-sectional study. Ann Intern Med.

[21] Bates J.E., Howell R.M., Liu Q. (2019). Therapy-related cardiac risk in childhood cancer survivors: an analysis of the childhood cancer survivor study. J Clin Oncol.

[22] Howell R.M., Giebeler A., Koontz-Raisig W. (2012). Comparison of therapeutic dosimetric data from passively scattered proton and photon craniospinal irradiations for medulloblastoma. Radiat Oncol.

[23] Yoon M., Shin D.H., Kim J. (2011). Craniospinal irradiation techniques: a dosimetric comparison of proton beams with standard and advanced photon radiotherapy. Int J Radiat Oncol.

[24] Ehrhardt M.J., Leerink J.M., Mulder R.L. (2023). Systematic review and updated recommendations for cardiomyopathy surveillance for survivors of childhood, adolescent, and young adult cancer from the International Late Effects of Childhood Cancer guideline harmonization group. Lancet Oncol.

[25] Children’s Oncology Group (2023). Long-term Follow-Up guidelines for survivors of childhood, adolescent, and young adult cancers. Version 6.0. https://www.survivorshipguidelines.org/pdf/2025/COG_LTFU_Guidelines_Only_v6.pdf.

[26] Thavendiranathan P., Poulin F., Lim K.D., Plana J.C., Woo A., Marwick T.H. (2014). Use of myocardial strain imaging by echocardiography for the early detection of cardiotoxicity in patients during and after cancer chemotherapy. J Am Coll Cardiol.

[27] Kalam K., Otahal P., Marwick T.H. (2014). Prognostic implications of global LV dysfunction: a systematic review and meta-analysis of global longitudinal strain and ejection fraction. Heart.

[28] Armstrong G.T., Joshi V.M., Ness K.K. (2015). Comprehensive echocardiographic detection of treatment-related cardiac dysfunction in adult survivors of childhood cancer. J Am Coll Cardiol.

[29] Van Der Pal H.J., Van Dalen E.C., Van Delden E. (2012). High risk of symptomatic cardiac events in childhood cancer survivors. J Clin Oncol.

[30] Belzile-Dugas E., Eisenberg M.J. (2021). Radiation-induced cardiovascular disease: review of an underrecognized pathology. J Am Heart Assoc.

[31] Bates J.E., Shrestha S., Liu Q. (2023). Cardiac substructure radiation dose and risk of late cardiac disease in survivors of childhood cancer: a report from the childhood cancer survivor study. J Clin Oncol.

[32] Armenian S., Bhatia S. (2018). Predicting and preventing anthracycline-related cardiotoxicity. Am Soc Clin Oncol Educ Book.

[33] Heemelaar J.C., Speetjens F.M., Al Jaff A.A.M. (2023). Impact of age at diagnosis on cardiotoxicity in high-grade osteosarcoma and ewing sarcoma patients. JACC CardioOncol.

[34] Menezes K.M., Wang H., Hada M., Saganti P.B. (2018). Radiation matters of the heart: a mini review. Front Cardiovasc Med.

[35] Dallaire F., Sarkola T., Kerkhof P.L.M., Miller V.M. (2018). Sex-specific analysis of cardiovascular function.

[36] Gebauer J., Higham C., Langer T., Denzer C., Brabant G. (2019). Long-term endocrine and metabolic consequences of cancer treatment: a systematic review. Endocr Rev.

[37] Chen Y., Chow E.J., Oeffinger K.C. (2020). Traditional cardiovascular risk factors and individual prediction of cardiovascular events in childhood cancer survivors. JNCI J Natl Cancer Inst.

[38] Clement S.C., Schouten-van Meeteren A.Y.N., Boot A.M. (2016). Prevalence and risk factors of early endocrine disorders in childhood brain tumor survivors: a nationwide, multicenter study. J Clin Oncol.

[39] Uday S., Murray R.D., Picton S. (2015). Endocrine sequelae beyond 10 years in survivors of medulloblastoma. Clin Endocrinol (Oxf).

[40] Van Santen H.M., Chemaitilly W., Meacham L.R., Tonorezos E.S., Mostoufi-Moab S. (2020). Endocrine health in childhood cancer survivors. Pediatr Clin North Am.

[41] Gurney J.G., Kadan-Lottick N.S., Packer R.J. (2003). Endocrine and cardiovascular late effects among adult survivors of childhood brain tumors: childhood cancer survivor study. Cancer.

[42] Armstrong G.T., Oeffinger K.C., Chen Y. (2013). Modifiable risk factors and major cardiac events among adult survivors of childhood cancer. J Clin Oncol.

[43] Pillai R., Zhang L., Peters K. (2025). Age- and sex-differences and reference values for ventricular strain by cardiovascular magnetic resonance imaging in adults without cardiovascular disease or cardiovascular disease risk factors. J Cardiovasc Magn Reson.

[44] Petitto M., Esposito R., Sorrentino R. (2018). Sex-specific echocardiographic reference values: the women’s point of view. J Cardiovasc Med.

[45] Tsang D.S., Patel S. (2019). Proton beam therapy for cancer. Can Med Assoc J.

