# Supplementary material for: Longitudinal Assessment of Cardiac Function After Craniospinal Irradiation in Pediatric Central Nervous System Tumor Survivors
Source: JACC Adv. 2026 Jun 15;5(7):102886. doi: 10.1016/j.jacadv.2026.102886 (PMC13285839; doi:10.1016/j.jacadv.2026.102886)

**Supplemental Appendix**

**Longitudinal Assessment of Cardiac Function After Craniospinal Irradiation in Pediatric Central Nervous System Tumor Survivors**

**Supplementary Table 1. Baseline Demographic and Clinical Characteristics of CNS Tumor Survivors With and Without Available Echocardiographic Data Pg. 2**

**Supplementary Table 2. Number of Echocardiographic Assessments per Patient Over Follow-up Pg. 5**

**Supplementary Table 3. Multivariable mixed-effects analyses of longitudinal shortening fraction and diastolic indices by clinical and treatment-related factors Pg. 6**

*Supplementary Table 3A. Left ventricular shortening fraction (M-mode). Pg. 6*

*Supplementary Table 3B. Mitral E/E’ lateral. Pg. 9*

*Supplementary Table 3C. Mitral E/A ratio. Pg. 12*

**Supplementary Figure 1. Trajectory of Global Longitudinal Strain After Radiation Therapy by Institution. Pg. 16**

**Supplementary Figure 2. Individual Longitudinal Trajectories of Echocardiographic Parameters Following Radiation Therapy. Pg. 17**

**Supplementary Table 1. Baseline Demographic and Clinical Characteristics of CNS Tumor Survivors With and Without Available Echocardiographic Data**

| **Demographic/Clinical characteristics** | **Overall (n=187)** | **With Echo Data (n=129)** | **Without Echo Data (n=58)** | **P-value** |
| --- | --- | --- | --- | --- |
| **Sex** |  |  |  | 1.00 |
| Female | 77 (41%) | 53 (41%) | 24 (41%) |  |
| Male | 110 (59%) | 76 (59%) | 34 (59%) |  |
| **Age at diagnosis (years)** | 8 (5-10) | 8 (5-11) | 7 (5-10) | 0.25 |
| **Current age (years)** | 22 (17-27) | 23 (18-27) | 20 (15-28) | 0.061 |
| **Diagnosis** |  |  |  | 0.012 |
| Atypical teratoid/rhabdoid tumor | 4 (2%) | 2 (2%) | 2 (3%) |  |
| CNS Hodgkin lymphoma | 1 (1%) | 0 | 1 (2%) |  |
| Embryonal Rhabdomyosarcoma | 1 (1%) | 1 (1%) | 0 |  |
| Ependymoma | 14 (7%) | 4 (3%) | 10 (17%) |  |
| Germinoma | 1 (1%) | 1 (1%) | 0 |  |
| Medulloblastoma | 141 (75%) | 103 (80%) | 38 (66%) |  |
| NGGCT | 6 (3%) | 4 (3%) | 2 (3%) |  |
| Oligodendroglioma spinal cord | 1 (1%) | 1 (1%) | 0 |  |
| Pineoblastoma | 12 (6%) | 10 (8%) | 2 (3%) |  |
| PNET | 6 (3%) | 3 (2%) | 3 (5%) |  |
| **Disease stage** | 170 | 123 | 47 | 0.036 |
| Average risk | 102 (60%) | 80 (65%) | 22 (47%) |  |
| High risk | 68 (40%) | 43 (35%) | 25 (53%) |  |
| **CSI as first-line therapy** | 187 | 129 | 58 | <0.001 |
| Yes | 166 (89%) | 124 (96%) | 42 (72%) |  |
| At relapse | 21 (11%) | 5 (4%) | 16 (28%) |  |
| **Radiation** | | | | |
| **Radiotherapy location** | 187 | 129 | 58 | 1.00 |
| Craniospinal | 185 (99%) | 127 (98%) | 58 (100%) |  |
| Spine only | 2 (1%) | 2 (2%) | 0 |  |
| **Radiation modality** | 187 | 129 | 58 | 0.74 |
| Photon | 176 (94%) | 122 (95%) | 54 (93%) |  |
| Proton | 11 (6%) | 7 (5%) | 4 (7%) |  |
| **CSI dose (cGy)** | 2,340 (2,340–3,600) | 2,340 (2,340–3,600) | 2,340 (2,340–3,600) | 0.19 |
| **Mean heart dose (cGy)** | 1,187.8 (1,040.8–1,543.5) | 1,216.9 (1,086.3–1,615.9) | 1,001.0 (756.0–1,236.0) | 0.004 |
| **Heart D50 (cGy)** | 1,491.2 (1,209.1–2,015.9) | 1,510.0 (1,412.0–2,016.2) | 898.0 (722.0–1,452.0) | <0.001 |
| **Chemotherapy** | | | | |
| Any chemotherapy | 177 (95%) | 124 (96%) | 53 (91%) | 0.29 |
| Vinca alkaloids | 162 (87%) | 118 (91%) | 44 (76%) | 0.005 |
| Cisplatin | 162 (87%) | 118 (91%) | 44 (76%) | 0.005 |
| Cyclophosphamide | 134 (72%) | 102 (79%) | 32 (55%) | 0.001 |
| Ifosfamide | 7 (4%) | 4 (3%) | 3 (5%) | 0.68 |
| Autologous HSCT | 71 (38%) | 64 (50%) | 7 (12%) | <0.001 |
| **Cardiac events during treatment** | 22 (12%) | 21 (16%) | 1 (2%) | 0.003 |
| **Comorbidities** |  |  |  | <0.001 |
| Endocrine | 108 (58%) | 87 (67%) | 21 (36%) | <0.001 |
| Cardiovascular | 39 (21%) | 36 (28%) | 3 (5%) | <0.001 |
| **Cardiac medication use** | 3 (2%) | 3 (2%) | 0 | 0.55 |

*Abbreviations: CNS: central nervous system; CSI: craniospinal irradiation; HSCT: hematopoietic stem cell transplantation*

**Supplementary Table 2. Number of Echocardiographic Assessments per Patient by Echocardiographic Parameter**

| **Number of Echoes** | **Overall Cohort (n=129)** | **M-Mode LVSF (n=128)** | **M-Mode LVEF (n=119)** | **GLS (n=100)** | **MVE/e′ Lateral Ratio (n=122)** | **E/A Ratio (n=117)** |
| --- | --- | --- | --- | --- | --- | --- |
| 1 | 43 (33.3%) | 42 (32.8%) | 46 (38.7%) | 44 (44.0%) | 39 (32.0%) | 39 (33.3%) |
| 2 | 31 (24.0%) | 32 (25.0%) | 28 (23.5%) | 20 (20.0%) | 32 (26.2%) | 29 (24.8%) |
| 3 | 19 (14.7%) | 18 (14.1%) | 17 (14.3%) | 16 (16.0%) | 17 (13.9%) | 21 (17.9%) |
| 4+ | 36 (27.9%) | 36 (28.1%) | 28 (23.5%) | 20 (20.0%) | 34 (27.9%) | 28 (23.9%) |

*Abbreviations: LVSF: left ventricular shortening fraction; LVEF: left ventricular ejection fraction; GLS: global longitudinal strain; MV E/E’: mitral valve E to e’ ratio; E/A ratio: early to late mitral inflow velocity ratio*

**Supplementary Table 3. Multivariable mixed-effects analyses of longitudinal shortening fraction and diastolic indices by clinical and treatment-related factors.**

*Supplementary Table 3A. Left ventricular shortening fraction (M-mode)*

| **Main variable** | **Model**  * | **Cyclophosphamide cumulative dose β (95% CI)** | **p** | **Mean heart dose β (95% CI)** | **p** | **Male β (95% CI)** | **p** | **Cardiac event during treatment β (95% CI)** | **p** | **Endocrine comorbidities β (95% CI)** | **p** | **Cardiovascular comorbidities β (95% CI)** | **p** | **Age at diagnosis β (95% CI)** | **p** |
| --- | --- | --- | --- | --- | --- | --- | --- | --- | --- | --- | --- | --- | --- | --- | --- |
| **Age at diagnosis** | **T** | -0.06 [-0.17, 0.04] | 0.24 | -0.19 [-0.88, 0.50] | 0.59 | 0.03 [-1.14, 1.20] | 0.96 | -0.21 [-1.67, 1.25] | 0.78 | -0.30 [-2.02, 1.41] | 0.73 | -0.22 [-1.56, 1.11] | 0.74 |  |  |
|  | **A** | -0.06 [-0.17, 0.05] | 0.32 | -0.18 [-0.91, 0.56] | 0.64 | 0.11 [-1.13, 1.35] | 0.86 | -0.22 [-1.81, 1.36] | 0.78 | -0.18 [-1.97, 1.61] | 0.84 | -0.08 [-1.50, 1.35] | 0.91 |  |  |
| **Cyclophosphamide cumulative dose** | **T** |  |  | -0.31 [-1.07, 0.44] | 0.42 | -0.12 [-1.34, 1.10] | 0.85 | 0.35 [-1.25, 1.94] | 0.67 | -0.10 [-1.83, 1.64] | 0.91 | -0.55 [-1.88, 0.78] | 0.42 | -0.27 [-0.46, -0.08] | 0.007 |
|  | **A** |  |  | -0.13 [-0.85, 0.59] | 0.72 | 0.11 [-1.06, 1.28] | 0.85 | -0.10 [-1.66, 1.45] | 0.90 | -0.24 [-1.95, 1.46] | 0.78 | -0.47 [-1.75, 0.81] | 0.47 | -0.06 [-0.28, 0.16] | 0.60 |
| **Mean Heart Dose** | **T** | -0.07 [-0.17, 0.04] | 0.23 |  |  | -0.06 [-1.30, 1.18] | 0.92 | 0.04 [-1.52, 1.59] | 0.96 | 0.17 [-1.56, 1.89] | 0.85 | -0.19 [-1.54, 1.16] | 0.78 | -0.29 [-0.49, -0.10] | 0.004 |
|  | **A** | -0.04 [-0.14, 0.07] | 0.49 |  |  | 0.13 [-1.09, 1.36] | 0.83 | -0.03 [-1.55, 1.50] | 0.97 | -0.31 [-2.05, 1.43] | 0.73 | -0.35 [-1.71, 1.01] | 0.61 | -0.11 [-0.34, 0.13] | 0.37 |
| **Sex** | **T** | -0.05 [-0.16, 0.06] | 0.37 | -0.39 [-1.12, 0.35] | 0.30 | -1.01 [-3.20, 1.18] | 0.37 | -0.08 [-1.65, 1.49] | 0.92 | 0.06 [-1.69, 1.82] | 0.94 | -0.48 [-1.85, 0.89] | 0.50 | -0.30 [-0.50, -0.10] | 0.004 |
|  | **A** | -0.03 [-0.14, 0.07] | 0.54 | -0.39 [-1.10, 0.32] | 0.28 | -0.82 [-5.43, 3.79] | 0.73 | 0.14 [-1.39, 1.68] | 0.86 | -0.29 [-2.03, 1.45] | 0.74 | -0.37 [-1.72, 0.98] | 0.59 | -0.14 [-0.37, 0.10] | 0.25 |
| **Cardiac event during treatment** | **T** | -0.05 [-0.16, 0.06] | 0.35 | -0.37 [-1.10, 0.37] | 0.33 | 0.06 [-1.19, 1.31] | 0.92 | -0.68 [-3.29, 1.93] | 0.61 | 0.15 [-1.60, 1.90] | 0.87 | -0.34 [-1.71, 1.04] | 0.63 | -0.30 [-0.50, -0.11] | 0.003 |
|  | **A** | -0.03 [-0.13, 0.07] | 0.57 | -0.40 [-1.10, 0.29] | 0.26 | 0.18 [-1.02, 1.38] | 0.77 | -4.27 [-8.98, 0.44] | 0.077 | -0.70 [-2.44, 1.03] | 0.43 | -0.38 [-1.69, 0.94] | 0.58 | -0.17 [-0.40, 0.05] | 0.135 |
| **Endocrine comorbidities** | **T** | -0.06 [-0.17, 0.04] | 0.23 | -0.37 [-1.07, 0.34] | 0.31 | 0.09 [-1.12, 1.30] | 0.88 | -0.20 [-1.71, 1.31] | 0.80 | -0.50 [-3.05, 2.06] | 0.70 | -0.62 [-1.94, 0.70] | 0.36 | -0.26 [-0.45, -0.07] | 0.010 |
|  | **A** | -0.04 [-0.14, 0.07] | 0.52 | -0.42 [-1.14, 0.30] | 0.26 | 0.10 [-1.14, 1.35] | 0.87 | 0.36 [-1.28, 1.99] | 0.67 | 3.62 [-3.02, 10.25] | 0.29 | -0.39 [-1.74, 0.97] | 0.58 | -0.16 [-0.40, 0.09] | 0.21 |
| **Cardiovascular comorbidities** | **T** | -0.05 [-0.16, 0.06] | 0.37 | -0.43 [-1.16, 0.30] | 0.25 | 0.08 [-1.17, 1.32] | 0.91 | 0.05 [-1.51, 1.61] | 0.95 | 0.13 [-1.62, 1.88] | 0.89 | -0.32 [-2.78, 2.13] | 0.80 | -0.29 [-0.49, -0.10] | 0.004 |
|  | **A** | -0.03 [-0.14, 0.08] | 0.58 | -0.41 [-1.12, 0.30] | 0.26 | 0.05 [-1.19, 1.28] | 0.94 | 0.08 [-1.47, 1.64] | 0.92 | -0.35 [-2.08, 1.38] | 0.69 | -2.99 [-9.68, 3.70] | 0.38 | -0.15 [-0.38, 0.07] | 0.188 |

*β coefficients represent adjusted associations from multivariable linear mixed-effects models with patient-level random intercepts. Two time scales were used: T = time since craniospinal irradiation and A = attained age (years). Time was modeled using natural cubic splines. All models account for repeated measures and adjust for the covariates shown in each column. Cyclophosphamide dose and mean heart dose are continuous variables; sex and clinical comorbidities are binary (reference: female and absence of condition, respectively). 95% confidence intervals are shown in brackets; p-values are two-sided.*

*Supplementary Table 3B. Mitral E/E’ lateral*

| **Main variable** | **Model**  * | **Cyclophosphamide cumulative dose β (95% CI)** | **p** | **Mean heart dose β (95% CI)** | **p** | **Male β (95% CI)** | **p** | **Cardiac event during treatment β (95% CI)** | **p** | **Endocrine comorbidities β (95% CI)** | **p** | **Cardiovascular comorbidities β (95% CI)** | **p** | **Age at diagnosis β (95% CI)** | **p** |
| --- | --- | --- | --- | --- | --- | --- | --- | --- | --- | --- | --- | --- | --- | --- | --- |
| **Age at diagnosis** | **T** | -0.02 [-0.07, 0.03] | 0.39 | 0.49 [0.19, 0.79] | 0.002 | 0.29 [-0.23, 0.81] | 0.28 | 0.94 [0.29, 1.59] | 0.006 | -0.39 [-1.08, 0.29] | 0.26 | -0.06 [-0.67, 0.55] | 0.84 |  |  |
|  | **A** | -0.02 [-0.07, 0.02] | 0.37 | 0.49 [0.19, 0.79] | 0.002 | 0.29 [-0.23, 0.82] | 0.27 | 0.90 [0.24, 1.56] | 0.009 | -0.33 [-1.02, 0.36] | 0.35 | -0.11 [-0.73, 0.51] | 0.73 |  |  |
| **Cyclophosphamide cumulative dose** | **T** |  |  | 0.63 [0.30, 0.95] | <0.001 | 0.32 [-0.20, 0.84] | 0.23 | 0.79 [0.11, 1.46] | 0.026 | -0.49 [-1.17, 0.20] | 0.165 | -0.03 [-0.61, 0.54] | 0.91 | -0.15 [-0.23, -0.07] | <0.001 |
|  | **A** |  |  | 0.66 [0.33, 0.99] | <0.001 | 0.31 [-0.22, 0.84] | 0.25 | 0.83 [0.14, 1.53] | 0.022 | -0.50 [-1.20, 0.20] | 0.166 | 0.05 [-0.55, 0.64] | 0.88 | -0.14 [-0.24, -0.05] | 0.003 |
| **Mean Heart Dose** | **T** | -0.02 [-0.06, 0.02] | 0.28 |  |  | 0.27 [-0.19, 0.73] | 0.26 | 0.82 [0.25, 1.40] | 0.007 | -0.53 [-1.14, 0.08] | 0.091 | -0.11 [-0.62, 0.40] | 0.68 | -0.17 [-0.24, -0.10] | <0.001 |
|  | **A** | -0.03 [-0.07, 0.01] | 0.21 |  |  | 0.24 [-0.23, 0.71] | 0.32 | 0.87 [0.28, 1.46] | 0.005 | -0.54 [-1.17, 0.09] | 0.093 | -0.05 [-0.58, 0.49] | 0.87 | -0.17 [-0.26, -0.09] | <0.001 |
| **Sex** | **T** | -0.03 [-0.07, 0.02] | 0.26 | 0.48 [0.18, 0.78] | 0.002 | 0.26 [-0.53, 1.05] | 0.52 | 0.98 [0.33, 1.64] | 0.004 | -0.39 [-1.07, 0.29] | 0.26 | -0.14 [-0.72, 0.45] | 0.65 | -0.15 [-0.23, -0.07] | <0.001 |
|  | **A** | -0.02 [-0.07, 0.02] | 0.32 | 0.47 [0.18, 0.77] | 0.002 | 0.39 [-1.37, 2.15] | 0.66 | 1.02 [0.36, 1.67] | 0.003 | -0.46 [-1.14, 0.22] | 0.188 | -0.16 [-0.75, 0.43] | 0.60 | -0.14 [-0.23, -0.05] | 0.003 |
| **Cardiac event during treatment** | **T** | -0.02 [-0.07, 0.02] | 0.28 | 0.49 [0.19, 0.78] | 0.002 | 0.32 [-0.20, 0.83] | 0.23 | 0.79 [-0.14, 1.73] | 0.097 | -0.43 [-1.10, 0.24] | 0.21 | -0.12 [-0.70, 0.45] | 0.67 | -0.15 [-0.23, -0.07] | <0.001 |
|  | **A** | -0.02 [-0.06, 0.03] | 0.43 | 0.43 [0.14, 0.72] | 0.005 | 0.31 [-0.20, 0.82] | 0.24 | 1.95 [0.21, 3.69] | 0.029 | -0.43 [-1.10, 0.25] | 0.22 | -0.13 [-0.71, 0.45] | 0.66 | -0.15 [-0.24, -0.06] | 0.002 |
| **Endocrine comorbidities** | **T** | -0.03 [-0.07, 0.01] | 0.21 | 0.50 [0.21, 0.78] | <0.001 | 0.29 [-0.21, 0.79] | 0.26 | 0.96 [0.33, 1.59] | 0.004 | -1.05 [-1.96, -0.15] | 0.024 | -0.24 [-0.81, 0.32] | 0.40 | -0.13 [-0.21, -0.05] | 0.001 |
|  | **A** | -0.02 [-0.07, 0.02] | 0.32 | 0.46 [0.16, 0.76] | 0.003 | 0.34 [-0.19, 0.87] | 0.22 | 1.07 [0.38, 1.75] | 0.003 | -0.15 [-2.61, 2.32] | 0.91 | -0.15 [-0.74, 0.44] | 0.61 | -0.14 [-0.24, -0.05] | 0.004 |
| **Cardiovascular comorbidities** | **T** | -0.02 [-0.07, 0.02] | 0.27 | 0.48 [0.18, 0.77] | 0.002 | 0.33 [-0.18, 0.85] | 0.21 | 0.98 [0.34, 1.63] | 0.004 | -0.43 [-1.10, 0.24] | 0.21 | 0.10 [-0.80, 1.00] | 0.82 | -0.15 [-0.23, -0.07] | <0.001 |
|  | **A** | -0.02 [-0.06, 0.02] | 0.38 | 0.46 [0.16, 0.75] | 0.003 | 0.32 [-0.20, 0.83] | 0.23 | 1.04 [0.39, 1.69] | 0.002 | -0.49 [-1.16, 0.18] | 0.152 | -1.27 [-3.92, 1.38] | 0.35 | -0.15 [-0.24, -0.06] | 0.001 |

*β coefficients represent adjusted associations from multivariable linear mixed-effects models with patient-level random intercepts. Two time scales were used: T = time since craniospinal irradiation and A = attained age (years). Time was modeled using natural cubic splines. All models account for repeated measures and adjust for the covariates shown in each column. Cyclophosphamide dose and mean heart dose are continuous variables; sex and clinical comorbidities are binary (reference: female and absence of condition, respectively). 95% confidence intervals are shown in brackets; p-values are two-sided.*

*Supplementary Table 3C. Mitral E/A ratio*

| **Main variable** | **Model**  * | **Cyclophosphamide cumulative dose β (95% CI)** | **p** | **Mean heart dose β (95% CI)** | **p** | **Male β (95% CI)** | **p** | **Cardiac event during treatment β (95% CI)** | **p** | **Endocrine comorbidities β (95% CI)** | **p** | **Cardiovascular comorbidities β (95% CI)** | **p** | **Age at diagnosis β (95% CI)** | **p** |
| --- | --- | --- | --- | --- | --- | --- | --- | --- | --- | --- | --- | --- | --- | --- | --- |
| **Age at diagnosis** | **T** | 0.01 [-0.01, 0.02] | 0.59 | 0.05 [-0.07, 0.18] | 0.42 | 0.06 [-0.15, 0.26] | 0.60 | -0.15 [-0.42, 0.11] | 0.26 | 0.09 [-0.18, 0.37] | 0.51 | -0.16 [-0.40, 0.08] | 0.197 |  |  |
|  | **A** | 0.01 [-0.01, 0.02] | 0.48 | 0.05 [-0.07, 0.18] | 0.41 | 0.06 [-0.15, 0.26] | 0.59 | -0.16 [-0.42, 0.11] | 0.26 | 0.09 [-0.19, 0.37] | 0.52 | -0.15 [-0.39, 0.09] | 0.21 |  |  |
| **Cyclophosphamide cumulative dose** | **T** |  |  | 0.01 [-0.12, 0.14] | 0.88 | 0.07 [-0.13, 0.27] | 0.49 | -0.14 [-0.41, 0.13] | 0.32 | 0.14 [-0.14, 0.41] | 0.34 | -0.18 [-0.40, 0.05] | 0.134 | -0.01 [-0.04, 0.02] | 0.49 |
|  | **A** |  |  | 0.00 [-0.13, 0.14] | 0.94 | 0.05 [-0.16, 0.25] | 0.67 | -0.13 [-0.41, 0.15] | 0.38 | 0.12 [-0.16, 0.40] | 0.40 | -0.15 [-0.38, 0.09] | 0.23 | -0.02 [-0.06, 0.02] | 0.29 |
| **Mean Heart Dose** | **T** | 0.01 [-0.01, 0.03] | 0.34 |  |  | 0.05 [-0.15, 0.25] | 0.64 | -0.16 [-0.42, 0.11] | 0.26 | 0.10 [-0.17, 0.37] | 0.46 | -0.16 [-0.39, 0.07] | 0.176 | -0.01 [-0.04, 0.02] | 0.55 |
|  | **A** | 0.00 [-0.01, 0.02] | 0.60 |  |  | 0.03 [-0.17, 0.24] | 0.76 | -0.19 [-0.46, 0.09] | 0.185 | 0.14 [-0.13, 0.42] | 0.31 | -0.17 [-0.41, 0.07] | 0.167 | -0.01 [-0.05, 0.03] | 0.57 |
| **Sex** | **T** | 0.00 [-0.01, 0.02] | 0.77 | 0.04 [-0.08, 0.15] | 0.56 | 0.29 [-0.02, 0.60] | 0.064 | -0.13 [-0.39, 0.12] | 0.31 | 0.15 [-0.12, 0.41] | 0.28 | -0.14 [-0.37, 0.08] | 0.22 | -0.01 [-0.04, 0.02] | 0.56 |
|  | **A** | 0.00 [-0.01, 0.02] | 0.65 | 0.06 [-0.06, 0.19] | 0.30 | 0.35 [-0.36, 1.07] | 0.34 | -0.21 [-0.48, 0.05] | 0.119 | 0.14 [-0.13, 0.41] | 0.31 | -0.15 [-0.38, 0.07] | 0.192 | -0.01 [-0.04, 0.03] | 0.73 |
| **Cardiac event during treatment** | **T** | 0.00 [-0.01, 0.02] | 0.66 | 0.04 [-0.08, 0.16] | 0.51 | 0.05 [-0.15, 0.25] | 0.61 | -0.20 [-0.58, 0.18] | 0.31 | 0.11 [-0.16, 0.37] | 0.42 | -0.16 [-0.38, 0.07] | 0.171 | -0.01 [-0.04, 0.02] | 0.47 |
|  | **A** | 0.00 [-0.01, 0.02] | 0.68 | 0.06 [-0.06, 0.18] | 0.30 | 0.04 [-0.16, 0.23] | 0.73 | 0.16 [-0.59, 0.91] | 0.68 | 0.14 [-0.13, 0.40] | 0.31 | -0.15 [-0.38, 0.07] | 0.184 | -0.01 [-0.04, 0.03] | 0.67 |
| **Endocrine comorbidities** | **T** | 0.00 [-0.01, 0.02] | 0.68 | 0.04 [-0.08, 0.16] | 0.48 | 0.06 [-0.14, 0.26] | 0.57 | -0.16 [-0.42, 0.09] | 0.22 | 0.02 [-0.35, 0.39] | 0.91 | -0.16 [-0.38, 0.07] | 0.180 | -0.01 [-0.04, 0.02] | 0.52 |
|  | **A** | 0.00 [-0.01, 0.02] | 0.68 | 0.06 [-0.06, 0.18] | 0.32 | 0.04 [-0.16, 0.25] | 0.67 | -0.19 [-0.46, 0.08] | 0.170 | 0.05 [-1.02, 1.13] | 0.92 | -0.16 [-0.39, 0.07] | 0.179 | -0.01 [-0.04, 0.03] | 0.78 |
| **Cardiovascular comorbidities** | **T** | 0.00 [-0.01, 0.02] | 0.66 | 0.03 [-0.09, 0.15] | 0.61 | 0.06 [-0.14, 0.25] | 0.57 | -0.17 [-0.43, 0.09] | 0.197 | 0.11 [-0.15, 0.37] | 0.41 | -0.08 [-0.42, 0.27] | 0.67 | -0.01 [-0.04, 0.02] | 0.52 |
|  | **A** | 0.00 [-0.01, 0.02] | 0.61 | 0.06 [-0.06, 0.18] | 0.32 | 0.04 [-0.16, 0.24] | 0.67 | -0.19 [-0.45, 0.06] | 0.146 | 0.14 [-0.12, 0.40] | 0.30 | -0.55 [-1.59, 0.49] | 0.30 | -0.01 [-0.04, 0.03] | 0.62 |

*β coefficients represent adjusted associations from multivariable linear mixed-effects models with patient-level random intercepts. Two time scales were used: T = time since craniospinal irradiation and A = attained age (years). Time was modeled using natural cubic splines. All models account for repeated measures and adjust for the covariates shown in each column. Cyclophosphamide dose and mean heart dose are continuous variables; sex and clinical comorbidities are binary (reference: female and absence of condition, respectively). 95% confidence intervals are shown in brackets; p-values are two-sided.*

**Supplementary Figure 1. Trajectory of Global Longitudinal Strain After Radiation Therapy by Institution**

Individual longitudinal trajectories of GLS following radiation therapy, stratified by institution (UHN and SickKids). Each line represents a single patient, with GLS plotted against time (months post-radiation). Analyses were restricted to studies with available GLS data reflecting incomplete acquisition in earlier years. The figure illustrates within-patient changes over time and inter-individual variability. Abbreviations: GLS: global longitudinal strain


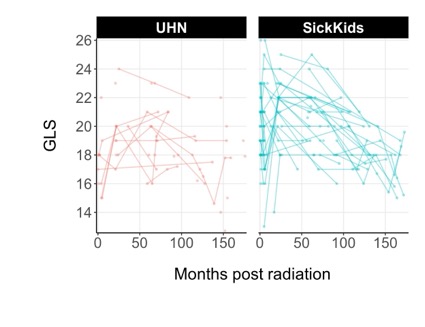


**Supplementary Figure 2. Individual Longitudinal Trajectories of Echocardiographic Parameters Following Radiation Therapy.**

Spaghetti plots depicting individual patient trajectories for echocardiographic parameters over time following radiation therapy. Each line represents a single patient, with values plotted against time (months post-radiation). These plots display the raw data, allowing direct visualization of within-patient changes and between-patient variability.


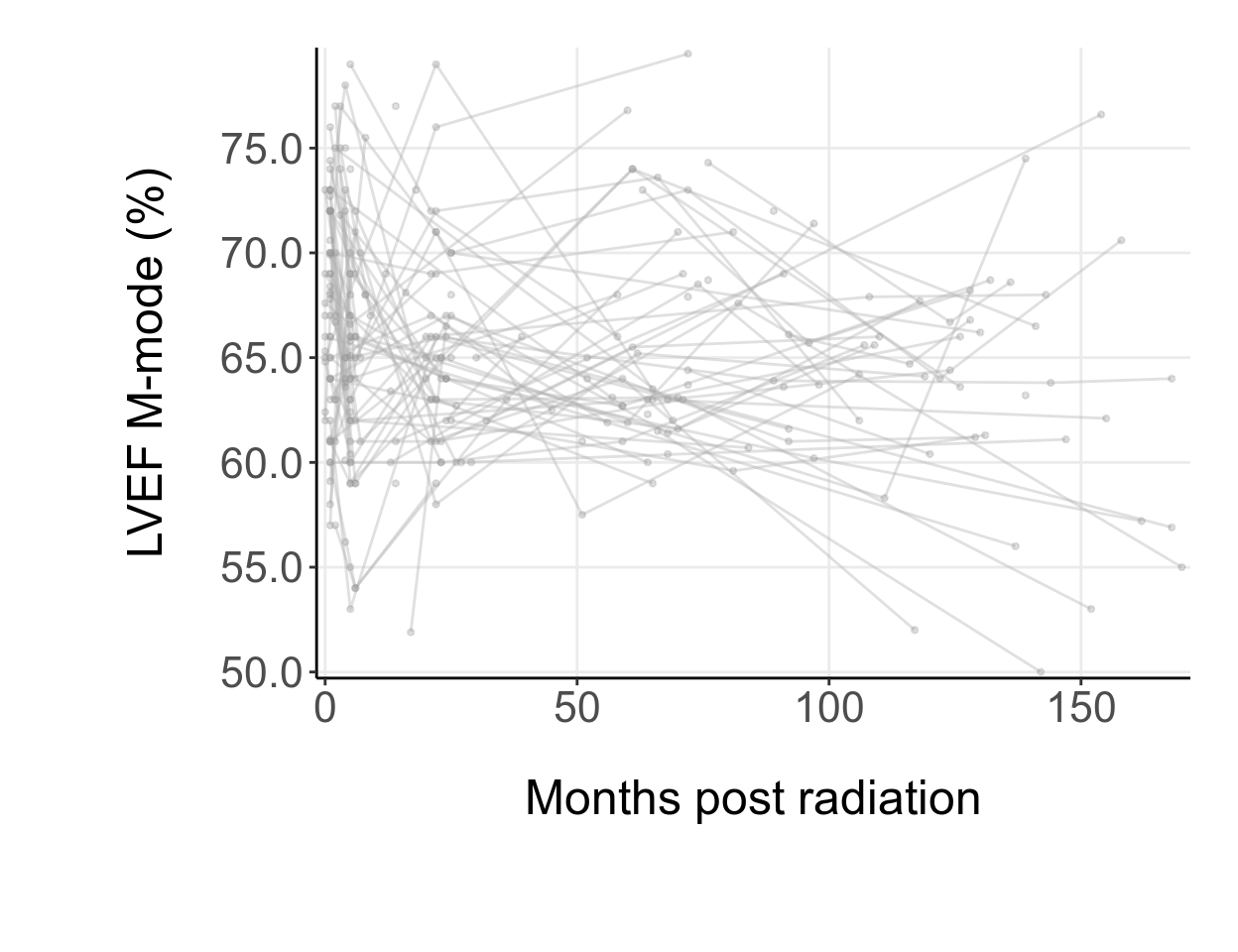


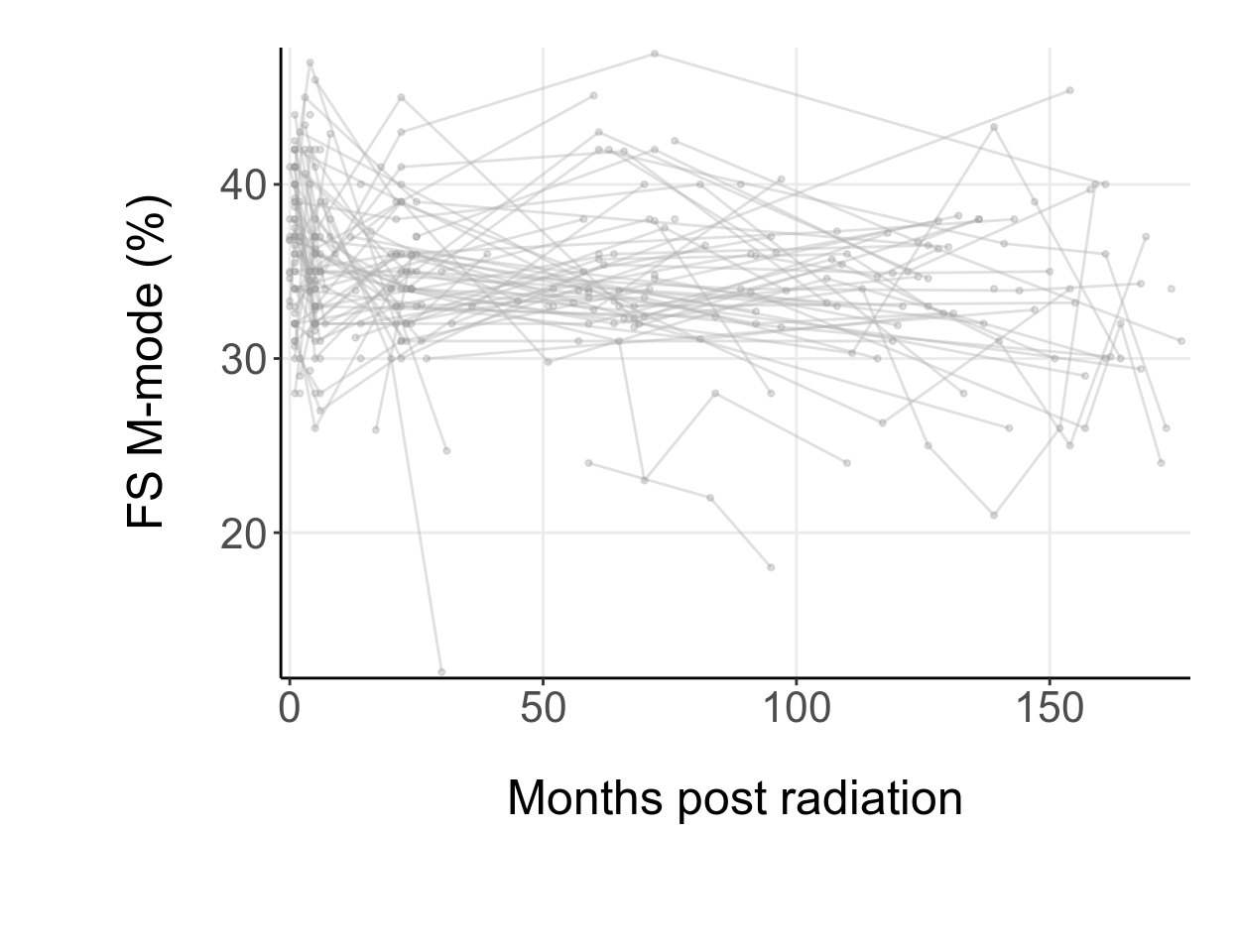


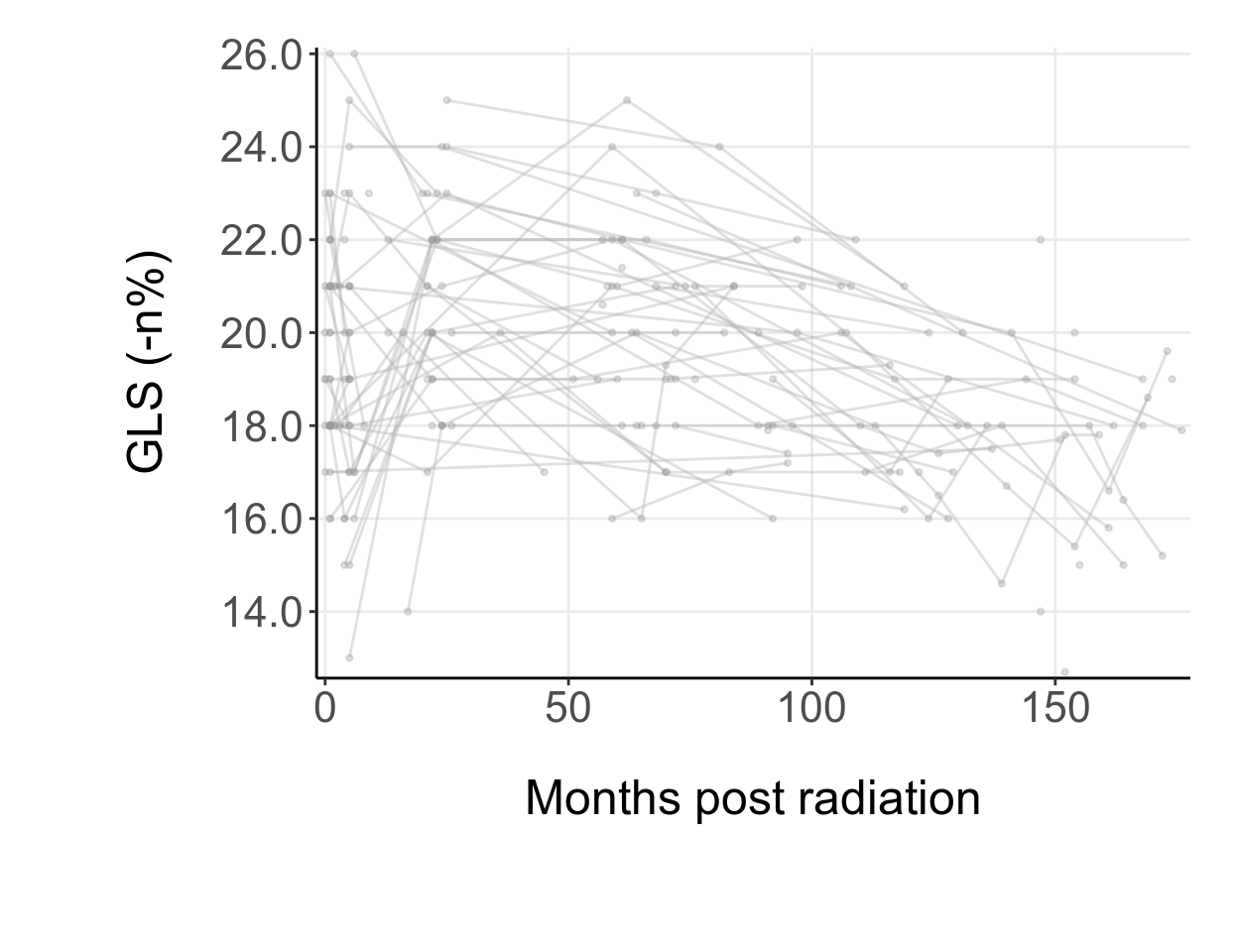


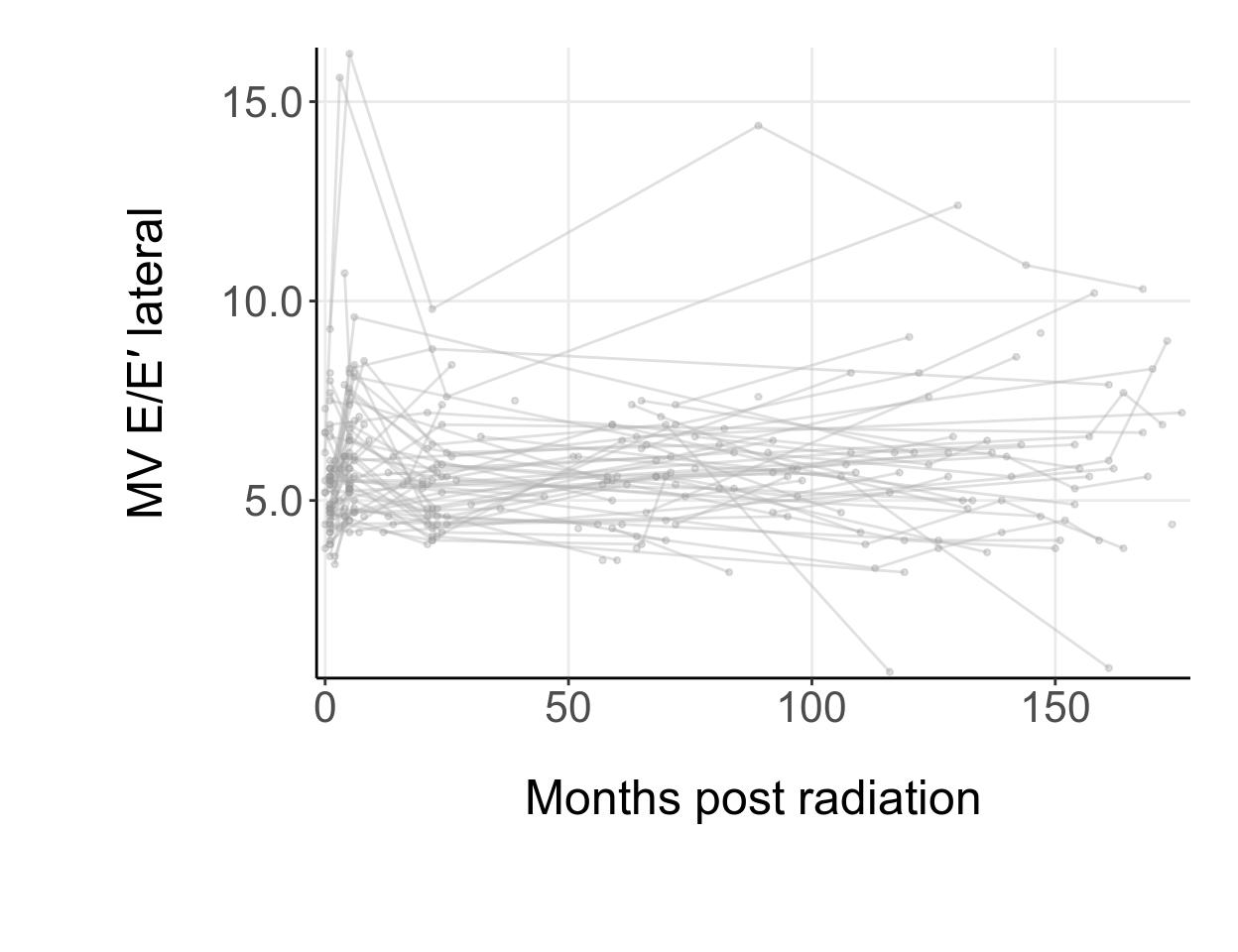


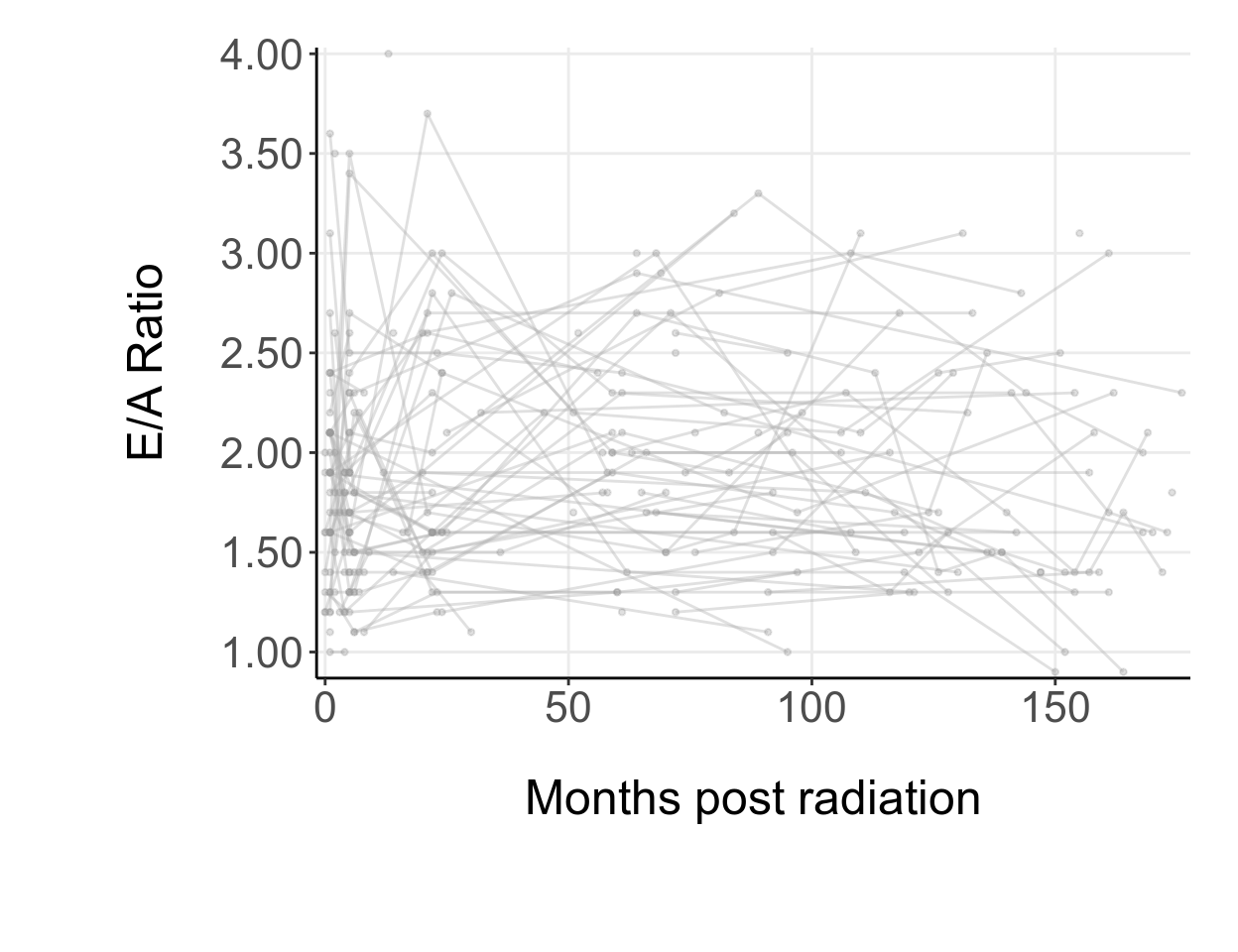

Supplement: Supplemental Material [file mmc1.docx]
